# Epigenetically poised chromatin states regulate *PRR* and *NLR* genes in soybean

**DOI:** 10.1007/s42994-025-00233-4

**Published:** 2025-08-08

**Authors:** Linzhe Jin, Yihan Zhang, Jiayuan Guo, Xuexia Liu, Yanling Lai, Xinfang Huang, Yuhan Zou, Shichuang Yan, Xianzhe Dai, Zhenhui Zhong

**Affiliations:** Ministry of Education Key Laboratory for Bio-Resource and Eco-Environment, College of Life Sciences, State Key Laboratory of Hydraulics and Mountain River Engineering, Sichuan University, Chengdu, 610064 China

**Keywords:** Epigenetic regulation, Pattern recognition receptors (PRR), Nucleotide-binding leucine-rich repeat (NLR), Soybean immunity, Chromatin poising

## Abstract

In the plant innate immune system, pattern recognition receptor (PRR) and nucleotide-binding domain leucine-rich repeat (NLR) proteins recognize pathogens and activate defenses. To prevent excessive immune responses that could affect growth, plants regulate PRRs and NLRs at the transcriptional and post-transcriptional levels. Poised or bivalent chromatin states, marked by the simultaneous presence of active and repressive epigenetic modifications, maintain genes in a transcriptionally primed state, keeping their expression low while enabling their rapid activation in response to stress. Here, we investigated how poised chromatin states regulate PRR and NLR genes in soybean (*Glycine max*). Our integrative epigenomic and transcriptomic analysis revealed that although NLR and PRR genes both harbor abundant active and repressive histone modifications and exhibit high chromatin accessibility, their basal expression levels remain relatively low. Moreover, clustered NLR and PRR genes residing within the same topologically associating domains shared similar chromatin states and expression dynamics, suggesting coordinated control. These gene families had distinct epigenetic features: NLR genes displayed narrow H3K27me3 peaks together with strong pausing of RNA Polymerase II at their 5′ ends, whereas PRR genes were characterized by broader H3K27me3 peaks. Together, our results shed light on the role of poised chromatin states in coordinating growth and defense responses in soybean.

**Supplementary Information:**

The online version contains supplementary material available at 10.1007/s42994-025-00233-4.

## Introduction

To combat the wide range of pathogens they encounter, plants have evolved complex immune systems consisting primarily of two layers of defense: pattern-triggered immunity (PTI) and effector-triggered immunity (ETI) (Jones and Dangl 2006; Li et al. 2020). PTI is activated when pattern recognition receptors (PRRs) detect conserved pathogen-associated molecular patterns, triggering defense responses such as mitogen-activated protein kinase cascades, calcium fluxes, and the production of reactive oxygen species. PRRs, such as lysine motif (LysM) receptors and FLAGELLIN-SENSITIVE 2 (FLS2), are crucial for early pathogen detection and their activities are tightly regulated to avoid inappropriate immune activation (Henry et al. 2013; Macho and Zipfel 2014). ETI, mediated by nucleotide-binding leucine-rich repeat (NLR) proteins, is a more specialized defense mechanism triggered by pathogen effectors (Cui et al. 2015). NLRs activate responses like the hypersensitive response, which limits pathogen spread. NLR genes are highly diverse, but their expression must be tightly controlled to prevent autoimmunity and fitness costs (Cui et al. 2015; Lai and Eulgem 2018). Indeed, overexpression of NLR genes such as *Pto* in tomato (*Solanum lycopersicum*) or *RESISTANT TO PSEUDOMONAS SYRINGAE4* (*RPS4*) in Arabidopsis (*Arabidopsis thaliana*) can induce cell death (Adachi et al. 2023; Huh 2022). Recent research also highlighted the existence of crosstalk between PTI and ETI, whereby PRRs cooperate with NLRs to enhance pathogen resistance (Yuan et al. 2021).

Soybean (*Glycine max*) is an important crop and a major source of protein and oil. Its growth is highly vulnerable to biotic stresses, with pathogen infections causing substantial yield losses and lower seed quality. Indeed, pathogen infections diminish annual soybean yields by an estimated 11% worldwide (Hartman et al. 1999). The major diseases that contribute significantly to lower global production are soybean rust (caused by *Phakopsora pachyrhizi* and *Phakopsora meibomiae*) (Goellner et al. 2010; Langenbach et al. 2016), root and stem rot (caused by *Phytophthora sojae* and *Fusarium* spp.) (Arias et al. 2013; Kamoun et al. 2015), and soybean cyst nematode disease (caused by *Heterodera glycines*) (Bent 2022; Lin et al. 2022). These pathogens compromise yield and seed viability, alter nutrient composition, and weaken overall plant health, severely threatening food security and agricultural sustainability. Understanding the molecular and epigenetic regulatory mechanisms underlying immune responses in soybean is therefore essential for developing durable disease resistance strategies, including genetic and/or epigenetic improvement and precision breeding approaches that enhance crop resilience without compromising growth or yield.

Poised chromatin states represent a key epigenetic mechanism by which gene expression can be maintained at a low level under normal conditions but rapidly activated in response to developmental or environmental cues (Lesch and Page 2014). Poised chromatin has a unique combination of epigenetic features: (1) bivalent chromatin modifications, with active histone marks (e.g., H3K4me3, H3K27ac) coexisting with repressive marks (e.g., H3K27me3) at promoters (Puri et al. 2015); (2) high chromatin accessibility; and (3) RNA polymerase II (Pol II) pausing at promoter-proximal regions (Gaertner et al. 2012; Gaertner and Zeitlinger 2014). Although poised chromatin has been extensively studied in animals, recent research has associated it with stress-responsive gene expression in plants (Gao et al. 2023; Qian et al. 2018). Poised chromatin may be particularly critical for immune genes, such as PRR and NLR genes, which require rapid yet tightly controlled activation to defend against pathogens without compromising plant fitness. Moreover, PRR and NLR genes frequently form gene clusters (Barragan and Weigel 2021; Graham et al. 2002; Shiu et al. 2004), which could facilitate the coordinated regulation of their expression by epigenetic mechanisms. However, the specific contribution of poised chromatin to the transcriptional regulation of PRR and NLR genes in plant immunity remains largely unexplored, especially in economically important crops like soybean.

In this study, we hypothesized that poised chromatin at PRR and NLR genes enables the rapid transcriptional activation required for an effective defense response against pathogens upon pathogen recognition in soybean while preventing unnecessary immune activation under normal conditions. To test this hypothesis, we integrated multiple global approaches, combining epigenomic profiling via chromatin immunoprecipitation sequencing (ChIP-seq), assay for transposase-accessible chromatin using sequencing (ATAC-seq), and high-throughput chromosome conformation capture (Hi-C), with transcriptome sequencing (RNA-seq) analysis to investigate the epigenetic regulation of PRR and NLR genes in soybean immunity. Our findings reveal that a substantial proportion of PRR and NLR genes are organized into genomic clusters enriched for bivalent chromatin modifications and higher chromatin accessibility, maintaining them in a poised state that enables their rapid activation upon infection. These insights provide a deeper understanding of how epigenetic mechanisms regulate plant immune responses.

## Results

### Exploration of the chromatin states for plant disease resistance (*R*) genes in soybean

**Fig. 1:**
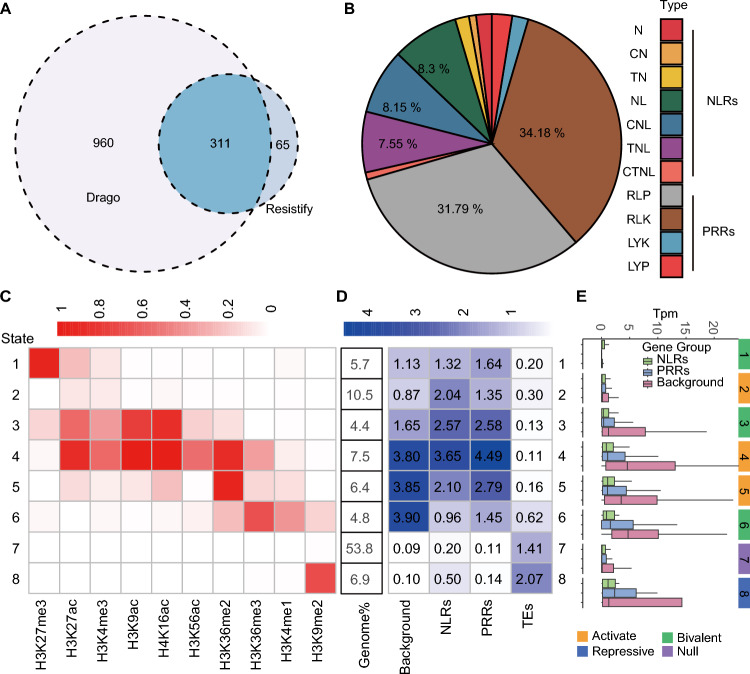
Epigenome profiling and chromatin states in soybean.** A** Venn diagram showing the extent of overlap between PRR genes and NLR genes identified by Drago3 and Resistify. **B** Detailed classification of PRRs and NLRs encoded by the soybean genome. N, NB-ARC domain; C, coiled-coil domain; T, TIR domain; L, LRR domain; RLP, receptor-like protein; RLK, receptor-like kinase; LYK, LysM receptor-like kinase; LY: LysM receptor-like protein. **C** Matrix showing the eight possible chromatin states of the soybean genome, using ChromHMM. Chromatin states were defined based on combinatorial patterns of histone modifications, identified through chromatin segmentation analysis. Each state represents a functional chromatin environment, such as active, poised, or repressed regions.** D** Analysis of the enrichment between background genes, NLR genes, PRR genes, and transposable elements (TEs) with the eight detected chromatin states. Fold enrichment was calculated as the ratio of the number of observed genes in each chromatin state to the expected number based on randomly selected gene sets of the same size. A fold enrichment greater than 1 indicates overrepresentation, with higher values corresponding to stronger enrichment. **E** Expression level of background genes, NLR genes, and PRR genes in different chromatin states

Emerging evidence suggests that epigenetic mechanisms, particularly histone modifications, may regulate the balance between immune responses and growth by modulating the transcriptional readiness of R genes. To explore this idea, we first annotated all R genes in the soybean genome, using the cultivar Williams 82 (Wm82), by a combination of the Pathogen Receptor Genes Database (PRGdb) and the bioinformatics tool Resistify (Sanseverino et al. 2010; Smith et al. 2025). We identified 1336 putative R genes, of which 394 are NLR genes and 942 are PRR genes (Fig. 1A and B). To define the chromatin states of these R genes, we collated publicly available ChIP-seq datasets for the histone modifications H3K4me3, H3K27me3, H3K36me3, H3K56ac, H3K4me1, and H3K27ac obtained from soybean leaf tissue and combined them with our own generated ChIP-seq datasets for H3K36me2, H3K9ac and H4K16ac, each representing two highly similar biological replicates (Fig. S1A). We mapped all clean reads from each dataset to the Wm82 reference genome, after which we determined their genomic context. The resulting distribution patterns were consistent with those observed in other plant species (Fig. S1B). We then employed the algorithm ChromHMM (Ernst and Kellis 2017) to integrate these multiple histone modification datasets and globally classify chromatin states.

We detected eight chromatin states across the genome, defining regions as being actively transcribed, poised for activation, or repressed (Fig. 1C). We observed that although some PRR genes and NLR genes are enriched in the active chromatin state (state 2), their expression levels remained relatively low (Fig. 1D). Notably, PRR genes and NLR genes were both significantly enriched among the genes in poised chromatin states (states 1 and 3), characterized by the coexistence of active (e.g., H3K4me3 or H3K acetylation) and repressive (e.g., H3K27me3) histone marks, indicating that these genes may be maintained in a transcriptionally "ready" state (Fig. 1D). Additionally, PRR and NLR genes were enriched among genes in state 4, which was dominated by active marks such as H3K27ac, H3K4me3, H3K9ac, H4K16ac, H3K36me2/3, and H3K56ac (Fig. 1D). Despite this enrichment, however, their expression levels remained lower than those of randomly selected control genes (Fig. 1E, Fig. S1C). This observation suggests that even within active chromatin environments, additional regulatory mechanisms may suppress basal expression of these immunity receptor genes under non-stress conditions. Overall, our genome-wide analysis provides a comprehensive view of the chromatin states associated with PRR genes and NLR genes in soybean.

### Transcriptional poising and chromatin landscape of *R* genes

To evaluate the transcriptional activity of R genes, we performed RNA polymerase II (Pol II) ChIP-seq and ATAC-seq experiments. These assays enabled us to assess Pol II occupancy (a key indicator of transcriptional readiness) and chromatin accessibility at R genes. For Pol II ChIP-seq, we used an antibody recognizing the C-terminal domain sequence YSPTSPS of Pol II phosphorylated at residue Ser-2, a hallmark of transcriptional elongation. In parallel, ATAC-seq provided information about the regions of open chromatin that are permissive to transcription factor binding.

**Fig. 2:**
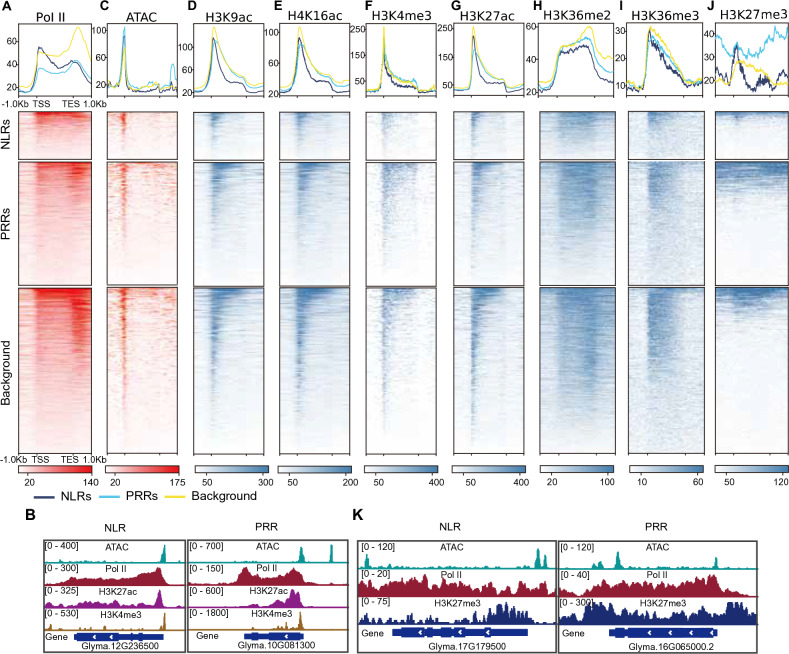
Transcriptional poising and chromatin landscape of R genes. **A** Metaplot (top) and heatmaps (bottom) representing ChIP-seq signal intensity for RNA Polymerase II over NLR genes, PRR genes, and randomly selected genes. **B** Integrated genome viewer windows showing Pol II pausing at the 5’ end of the NLR gene *Glyma.12G23650* and the general Pol II pattern over the PRR gene Glyma.10G081300. **C–J** Metaplot (top) and heatmaps (bottom) representing signal intensity from ATAC-seq **(C)**, H3K9ac ChIP-seq **(D)**, H4K16ac ChIP-seq **(E)**, H3K4me3 ChIP-seq **(F)**, H3K27ac ChIP-seq **(G)**, H3K36me2 ChIP-seq **(H)**, H3K36me3 ChIP-seq **(I)**, and H3K27me3 ChIP-seq **(J)** data over NLR genes, PRR genes, and randomly selected genes. **K** Integrated genome viewer windows showing H3K27me3 enrichment at the 5’ end of the NLR gene Glyma.17G179500 and the PRR gene Glyma.16G065000

Our analysis revealed that NLR genes display a distinctive pattern: they exhibit pronounced Pol II pausing, characterized by higher occupancy of Ser-2P Pol II at the 5′ end of NLR genes and markedly lower occupancy at the 3′ end of NLR genes compared to randomly selected genes (Fig. 2A and B). This pattern indicates that NLR genes are maintained in a transcriptionally poised state, with Pol II predominantly recruited to promoters but not fully engaged in productive transcript elongation. By contrast, the distribution of Pol II along PRR genes was similar to that of randomly selected genes, albeit with overall lower occupancy (Fig. 2A and B). Furthermore, ATAC-seq data showed that chromatin accessibility at the transcription start sites (TSS) of PRR genes is slightly lower relative to that of randomly selected genes, whereas TSS accessibility was comparable for NLR genes. However, chromatin accessibility at the 3′ end of NLR genes was lower than that for randomly selected genes (Fig. 2C). We also profiled active histone modification marks (H3K9ac, H4K16ac, H3K4me3, H3K27ac, H3K36me2, H3K36me3) and the repressive mark H3K27me3 (Fig. 2D–J). Active marks were less enriched at the 5′ ends of PRR genes and NLR genes but showed lower enrichment over the gene bodies toward the 3′ ends of NLR genes (Fig. 2C–F). Conversely, the repressive mark H3K27me3 was more enriched over PRR gene and NLR genes than that seen for randomly selected genes, with PRR genes showing higher levels of H3K27me3 across gene bodies and flanking regions, but the enrichment was predominantly at the 5′ end of NLR genes (Fig. 2J and K).

To rule out the possibility that these patterns were merely due to variations in gene expression levels, we grouped PRR genes and NLR genes into groups with high (TPM ≥ 1) or low expression (TPM < 1). Metaplots of ATAC-seq, Pol II, and histone modification peaks across these groups revealed similar patterns (Fig. S2A–I). Together, our results demonstrate that the distinct chromatin landscapes of NLR genes and PRR genes may contribute to their differential transcriptional activities. In particular, the maintenance of NLR genes in a poised state, characterized by promoter-bound, paused Pol II, and a narrow H3K27me3 distribution, is very similar to the poised state required for rapid yet precise activation in response to pathogen attacks.

### *R* genes form clusters that are located in the same TAD domains

**Fig. 3:**
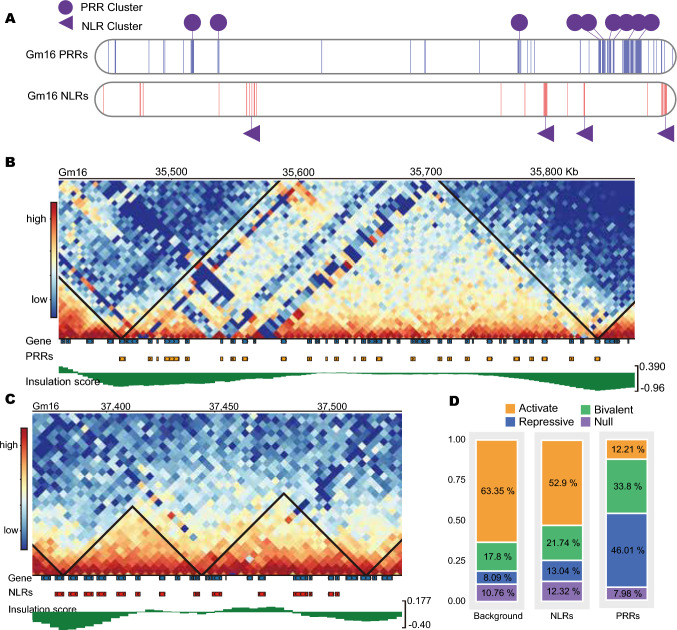
Spatial genomic organization of clusters of PRR genes and NLR genes and their chromatin contexts in soybean. **A** Location of R genes and clusters of R genes on chromosome 16 of the soybean genome. Blue, PRR genes; red, NLR genes. **B** High-resolution chromatin conformation capture (Hi-C) matrix showing the location of PRR gene clusters in topologically associating domains (TADs), indicated by triangles formed by diagonal lines. **C** Hi-C matrix showing that NLR gene clusters responsible for resistance to powdery mildew (*Rmd-c*) and *Phytophthora* stem and root rot (*Rps2*) are located in TADs (triangles). **D** Proportions of the four chromatin types for PRR genes and NLR genes located in TADs and background genes

Consistent with previous studies in Arabidopsis and rice (*Oryza sativa*), we also observed that R genes are not randomly distributed across the soybean genome but tend to form distinct clusters in specific chromosomal regions (Barragan and Weigel 2021; Shiu et al. 2004). Among the 942 PRR genes and 394 NLR genes annotated in our study, we identified 33 clusters of PRR genes and 26 clusters of NLR genes, encompassing 213 PRR genes and 138 NLR genes, respectively (Table S1). These clusters, which contain multiple R genes in close proximity, suggest a coordinated regulatory mechanism that may enhance the efficiency of stress response activation. We asked whether clusters were spatially linked within the three-dimensional (3D) genome space by integrating published Hi-C data into our datasets. This Hi-C analysis revealed that most NLR genes (68.1%: 94/138) and PRR genes (82.6%: 176/213) are co-localized within the same 3D genomic domains, forming interaction hubs that facilitate functional connectivity (Fig. S3A and B). This pattern was particularly prominent on chromosome 16 (Fig. 3A). Notably, the genomic regions containing NLR and PRR clusters often corresponded to topologically associating domains (TADs), which constrain chromatin interactions and regulate gene expression within their boundaries (Fig. 3B and C). For instance, we detected a previously reported cluster containing the *Resistance to powdery mildew (Rmd-c*) and *Phytophthora stem and root rot* (*Rps2*) genes in two adjacent TADs (Graham et al. 2002). Additionally, we determined that PRR genes within clusters are more likely to be enriched in bivalent or repressive modifications than NLR genes and randomly selected genes (Fig. 3D). The localization of R gene clusters within TADs suggests that chromatin architecture may play a role in their transcriptional co-regulation, potentially ensuring a rapid and coordinated immune response to biotic stress.

### TAD-dependent co-regulatory patterns of *R* gene clusters

**Fig. 4:**
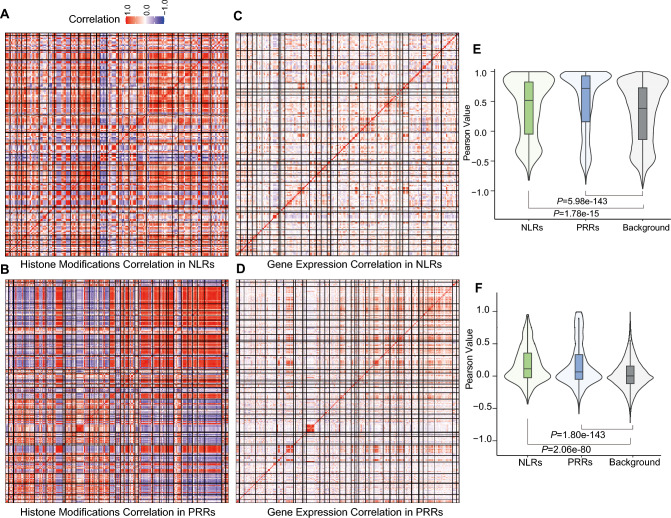
TAD-dependent co-regulatory patterns of NLR and PRR gene clusters as a function of their chromatin modifications and transcript levels. **A–D** Pairwise Pearson’s correlation coefficient analysis of histone modifications (**A, B**) and gene expression levels (**C, D**) for NLR gene clusters (**A, C**) and PRR gene clusters (**B, D**) across TADs. The black lines within each heatmap separate the TADs, for which Pearson’s correlation coefficients were calculated. **E** Distribution of pairwise Pearson’s correlation coefficients for histone modification levels of NLR genes (green), PRR genes (blue), and randomly selected background genes (gray). **F** Distribution of pairwise Pearson’s correlation coefficients for gene expression levels of NLR genes (green), PRR genes (blue), and randomly selected background genes (gray)

**Fig. 5:**
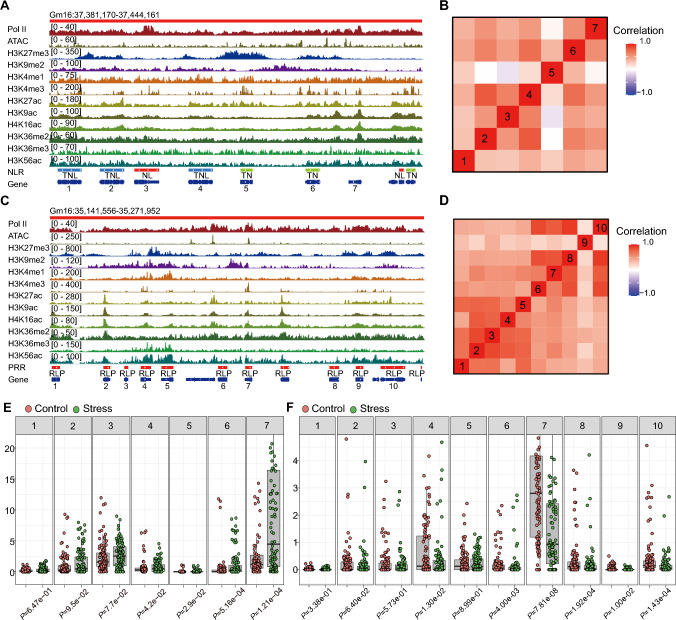
TAD-dependent co-regulatory patterns of NLR genes and PRR genes. **A** Representative integrated genome viewer windows illustrating cases of intra-TAD co-regulation showing high correlation of histone modification patterns among NLR genes within a TAD. **B** Gene expression correlation of NLR genes within the TAD. **C** Representative integrated genome viewer windows illustrating cases of intra-TAD co-regulation showing high correlation of histone modification patterns among PRRs within a TAD. **D** Correlation matrix between the expression levels of PRR genes within the TAD. **E** Expression levels of genes in the NLR cluster shown in (**A**) under normal and various stress conditions. **F** Expression levels of genes in the PRR cluster shown in (**C**) under normal and stress conditions

We observed that TADs containing R gene clusters were enriched in genes with similar chromatin states, suggesting a coordinated regulatory environment (Fig. 5A and C). To investigate this point in more detail, we calculated the Pearson correlation coefficient for histone modification levels in pairs of NLR genes or PRR genes across the genome and within TADs. We determined that NLR or PRR gene clusters within the same TAD exhibit significantly higher correlation coefficients for their histone modification patterns than randomly selected genes, suggesting that these genes share similar epigenetic landscapes within their respective TADs (Fig. 4A and B, Fig. S4A and B). Additionally, we noticed that different TADs are more likely to merge into larger TAD structures, particularly in the case of PRR genes, suggesting the potential existence of higher-order chromatin organization (Fig. 4B).

To examine the functional consequences of these chromatin modifications on gene expression, we calculated the pairwise Pearson’s correlation coefficient between pairs of NLR or PRR genes, using gene expression data. Specifically, we used a comprehensive published dataset of soybean leaf expression datasets collected under various biotic stresses, including infection with cucumber mosaic virus and fungal pathogens. From this analysis, we observed that NLR and PRR genes located within TADs are more likely to be co-expressed during biotic stress than those not located within TADs (Fig. 4C and D). Moreover, the Pearson correlation coefficients for histone modification levels and gene expression levels of NLR genes and PRR genes were significantly higher than those of randomly selected genes (Fig. 4E and F). For example, the NLR TAD containing the *Rmd-c* and *Rps2* genes was enriched with repressive and active histone marks (Fig. 5A and B). Genes within this cluster were highly co-expressed upon stress (Fig. 5E). The histone modification and gene expression profiles in another PRR cluster were also highly coordinated (Fig. 5C, D and F). We verified the expression dynamics of these two clusters under *Fusarium* treatment using RT-qPCR and observed a high correlation, supporting the robustness of our analysis (Supplementary Fig. S5). Together, these results highlight the potential role of TAD-based chromatin organization in facilitating coordinated immune responses.

## Discussion

The growing availability of sequenced genomes increasingly reveals R gene diversity across species and populations. Plant genomes encode a large repertoire of PRR and NLR immune receptors (the NLRome), which serve as a crucial innate defense mechanism against diverse pathogens. The expression and regulation of R genes must be tightly controlled to prevent excessive immune activation, which could otherwise lead to fitness costs. Multiple regulatory layers, including epigenetic mechanisms, protein–protein interactions with chaperones or helpers, and post-transcriptional modifications, fine-tune R gene expression and activation (Couto and Zipfel 2016; Cui et al. 2015; Tang et al. 2021; Zhang et al. 2024; Withers and Dong 2017). Additionally, some pathogen effectors actively manipulate R gene expression to suppress plant immunity (Wang et al. 2022), highlighting an ongoing host–pathogen evolutionary arms race. Here, we explored the epigenetic regulation of PRR genes and NLR genes in soybean. Our study reveals that PRR genes and NLR genes in soybean are characterized by a complex chromatin landscape, with the simultaneous enrichment of active and repressive histone modifications and high chromatin accessibility at their loci, while maintaining relatively low basal expression levels under normal conditions. Notably, these immune genes are frequently organized into clusters within the same TADs, which may facilitate their coordinated regulation. Furthermore, we identified distinct epigenetic features between the two gene families: NLR genes showed narrow H3K27me3 peaks and strong 5′ end Pol II pausing, whereas PRR genes had broader H3K27me3 peaks. Collectively, these findings suggest that a poised chromatin state is important for enabling the rapid, yet tightly controlled, activation of immune genes in soybean, thereby balancing growth and defense responses.

Epigenetic mechanisms, particularly DNA methylation and histone modifications, play a pivotal role in controlling R gene expression (Cambiagno et al. 2018; Xie and Duan 2023; Yang et al. 2020). Mutants defective in the RNA-directed DNA methylation (RdDM) pathway exhibit higher expression levels of R genes and altered immune responses, suggesting that DNA methylation contributes to the transcriptional repression of plant immune receptor genes in the absence of pathogens (Yu et al. 2013). Similarly, in rice, the *Pigm* locus comprises two antagonistic NLR genes: *PigmR*, which confers resistance, and *PigmS*, which suppresses immune activation to balance defense and yield. *PigmS* is specifically expressed in pollen but remains repressed at other developmental stages through the RdDM pathway (Deng et al. 2017). This epigenetic suppression prevents excessive energy expenditure for immunity under normal growth conditions while enabling transcriptional activation upon pathogen recognition. In addition to DNA methylation, histone modifications also play an essential role in the regulation of R gene expression. The establishment of a permissive or repressive chromatin state influences whether R genes remain transcriptionally poised or silenced. For instance, a study in Arabidopsis demonstrated that the NLR gene *SUPPRESSOR OF NPR1-1, CONSTITUTIVE 1* (*SNC1*) is tightly regulated by MODIFIER OF snc1 (MOS1) and DECREASED DNA METHYLATION 1 (DDM1) (Li et al. 2010). By contrast, the random insertion of a transgene containing the promoter and coding region of *SNC1* resulted in significantly higher transcript levels than the native *SNC1* copy, with H3K4me3 deposited on its promoter and activating its expression, highlighting the critical role of chromatin context in regulating NLR gene transcription (Binenbaum et al. 2025; Li et al. 2007). Our findings support this notion, showing that R gene clusters in soybean are enriched in genes in poised chromatin states, allowing for rapid transcriptional activation upon pathogen invasion while minimizing constitutive expression under normal conditions.

The clustering of PRR genes and NLR genes is a prevalent feature in plant genomes, resulting in rapid expansion and contraction of R gene families. This dynamic structural organization leads to presence/absence variation polymorphisms of R genes within a species, allowing for adaptive diversification of immunity genes (Barragan and Weigel 2021). For example, in rice, the *Piz* locus contains multiple blast resistance genes, including *Pi2*, *Pi9*, *Pi50*, *Pigm*, *Piz*, and *Piz-t*, whereas the *PiK* locus harbors *Pi1*, *Pik-h*, *Pik-m*, and *Pik-p*, highlighting the evolutionary advantage of NLR gene clustering (Qu et al. 2006; Zhou et al. 2006). Our findings reveal that R gene clusters are organized within TADs in the three-dimensional structure of the soybean genome. This spatial organization places the entire cluster under the control of similar chromatin modification marks, ensuring that these immune receptor genes are co-regulated in response to biotic stress. This spatial proximity within TADs may enable a more synchronized and rapid transcriptional response, ensuring efficient activation of R genes upon pathogen invasion.

We also observed differences in the chromatin landscapes of PRR genes versus NLR genes. The narrow H3K27me3 peaks observed at NLR genes suggest a highly localized repressive signal close to the TSS, which may facilitate the rapid removal of these marks upon pathogen detection. This precise repression is complemented by strong Pol II pausing at the 5′ end, ensuring that NLR genes are kept in a transcriptionally ready state that can be swiftly activated. By contrast, PRR genes exhibited broader H3K27me3 peaks, suggesting a more diffuse and stable repressive chromatin environment. Such wide peaks might contribute to a more robust basal repression of PRR gene expression, potentially requiring additional regulatory signals for their rapid induction. Notably, we also observed that TADs enriched with PRR genes are more likely to form higher-order chromatin structures, which would further influence their coordinated regulation. These distinct epigenetic features highlight how NLR genes are maintained in a highly dynamic, rapidly inducible state, whereas PRR genes are under a more robust repression, reflecting their differential kinetics in pathogen response and the need for precise regulation to balance growth and defense.

This study provides evidence supporting the epigenetic regulation of PRR genes and NLR genes and their poised chromatin states in plant immunity, but several limitations and future directions should be highlighted: (1) This study mainly focused on static chromatin states and did not monitor dynamic changes in chromatin or Pol II pausing during pathogen infection due to technical difficulties in capturing sufficient numbers of plant cells invaded by pathogens. Thus, a time-course analysis at the single-cell level could provide a more comprehensive view of how poised R genes transition to active states upon pathogen recognition. (2) The data were collected from leaves, limiting insights into the tissue-specific chromatin states of R genes. Given that immune responses can vary between tissues (Munch et al. 2018), future studies should use more tissues to explore how the regulation of R gene expression differs across different cell types. (3) This study identified bivalent chromatin states, high chromatin accessibility, and Pol II pausing as hallmarks of transcriptionally poised R genes; however, future functional validation is needed through mutational or epigenome-editing experiments to establish causality between these epigenetic states and R gene activation. (4) According to Flor’s gene-for-gene theory, the activation of some NLR genes may be dependent on the presence of a corresponding pathogen effector. However, we did not account for the fact that some poised NLR genes may not be activated in the absence of their specific pathogen-derived effectors. (5) This study was performed in soybean, and it remains unclear whether these findings apply to other plant species, particularly monocots such as rice and wheat (*Triticum aestivum*). Comparative epigenomic analysis across different crop species would be valuable in determining whether poised chromatin states are a conserved regulatory mechanism in plant immunity.

Collectively, our findings provide new insights into how epigenetic mechanisms and 3D genome organization contribute to the regulation of R gene expression in plants. The identification of poised chromatin states in clusters of PRR genes and NLR genes suggests that plants employ epigenetic priming as a regulatory strategy to maintain immune readiness while preventing autoimmunity. Understanding these mechanisms will be helpful for crop breeding strategies aimed at enhancing disease resistance without compromising yield. Future studies should explore how epigenetic reprogramming and chromatin architecture modifications can be leveraged to optimize PRR gene and NLR gene regulation for improved plant immunity and agricultural sustainability.

## Materials and methods

### Plant materials

The soybean cultivar Williams 82 (Wm82) was used as the plant material in this study. Plants were grown in a controlled greenhouse under a long-day (16 h light/8 h dark) photoperiod at a constant temperature of 26 °C to ensure optimal growth. Four-week-old soybean plants were selected for tissue collection to ensure a uniform developmental stage. Fully expanded leaves were harvested and immediately flash-frozen in liquid nitrogen.

### Annotation of soybean *R* genes

To gain a comprehensive and precise annotation of resistance (R) genes in the Wm82 soybean genome, a multifaceted approach was employed that integrates several bioinformatics tools and databases. First, the Plant Gene Resources Database (PGRDB) was used to identify and classify R genes based on conserved domains and motifs characteristic of known resistance proteins (Sanseverino et al. 2010). Then, Resistify, a tool leveraging machine learning algorithms trained on known plant NLRs, was applied to enhance the accuracy of our annotations (Smith et al. 2025). Protein hits from the two above approaches were merged to assemble the final dataset. PRR and NLR gene clusters were defined as gene clusters that contain at least three PRR or NLR genes, with up to three other genes inserted between two R genes.

### Chromatin immunoprecipitation sequencing (ChIP-seq) and analysis

For ChIP-seq, 1 g of leaf tissue was collected to investigate the binding profiles of Pol II (ab5059, Abcam, Ser-2 phosphorylated), as well as the enrichment of H3K36me2 (ab9049, Abcam), H3K9ac (ab4441, Abcam) and H4K16ac (ab109463, Abcam) modifications. ChIP experiments were performed following previously established protocols (Zhong et al. 2012). Briefly, 1 g of freshly collected leaf tissue from 4-week-old plants was ground in liquid nitrogen to obtain a fine powder. Chromatin was crosslinked with 1% (w/v) formaldehyde in nuclei isolation buffer for 10 min at room temperature to preserve protein–DNA interactions. The crosslinking reaction was terminated by the addition of freshly prepared 1.7 mL of 2 M glycine, followed by chromatin shearing using a Bioruptor Plus (Diagenode) to achieve fragment sizes of 200–500 bp. Immunoprecipitation was performed using the respective antibodies at 4 °C overnight, followed by incubation with Protein A and Protein G magnetic Dynabeads (Invitrogen) for 2 h at 4 °C. After immunoprecipitation, reverse crosslinking was carried out at 65 °C overnight, and protein–DNA complexes were treated with proteinase K (Invitrogen) at 45 °C for 4 h to remove proteins. Genomic DNA was purified by ethanol precipitation with 50 µL 3 M sodium acetate (Invitrogen), 2 µL GlycoBlue (Invitrogen), and 1 mL ethanol at − 20 °C overnight. The purified DNA was used for library preparation with an Ovation Ultra Low System V2 kit (NuGEN) following the manufacturer’s instructions. ChIP-seq libraries were sequenced on an Illumina NovaSeq platform. ChIP-seq data for H3K4me3, H3K27me3, H3K36me3, H3K56ac, H3K4me1, and H3K27ac modifications were obtained from previous studies (Lu et al. 2019; Ma et al. 2023; Wang et al. 2021). Adaptor sequences were removed from raw ChIP-seq reads using Trim Galore (https://github.com/FelixKrueger/TrimGalore). Trimmed reads were aligned to the Wm82 soybean reference genome (Chang S et al. 2013) using Bowtie2 (v2.1.0), allowing only uniquely mapped reads with no mismatches (Langmead and Salzberg 2012). Duplicate reads were removed using Samtools to minimize PCR amplification bias. Bigwig format track files were generated using the bamCoverage tool of deeptools (v3.5.1) with RPKM normalization for metaplots (Ramirez et al. 2016).

### ATAC-seq and analysis

Nuclei isolation from leaf tissues was performed following previously described protocols (Liu et al. 2018). Freshly isolated nuclei were immediately used for ATAC-seq following established methods (Buenrostro et al. 2013; Zhong et al. 2021). For nuclei extraction, about 1 g of leaf tissue was collected and immediately transferred into ice-cold grinding buffer containing 300 mM sucrose, 20 mM Tris–HCl pH 8.0, 5 mM MgCl_2_, 5 mM KCl, 0.2% (v/v) Triton X-100, 5 mM β-mercaptoethanol, and 35% (v/v) glycerol. The tissue was homogenized using an Omni International General Laboratory Homogenizer at 4 °C and filtered sequentially through two layers of Miracloth and a 40 µm nylon mesh cell strainer (Fisher). The filtrate was centrifuged at 3000 × *g* for 10 min at 4 °C, and the supernatant was discarded. The pellet was resuspended in 25 mL of fresh grinding buffer using a Dounce homogenizer, and the washing step was repeated twice to ensure high-purity nuclei. The isolated nuclei were resuspended in 0.5 mL freezing buffer (50 mM Tris–HCl pH 8.0, 5 mM MgCl_2_, 20% [v/v] glycerol, and 5 mM β-mercaptoethanol) and immediately subjected to Tn5 transposition. The transposition reaction was performed in a 50 µL reaction mixture, consisting of 25 µL 2 × dimethylformamide (DMF) buffer (66 mM Tris–acetate pH 7.8, 132 mM potassium acetate, 20 mM magnesium acetate, and 32% [v/v] DMF), 2.5 µL Tn5 transposase [Illumina]), and 22.5 µL nuclei suspension, incubated at 37 °C for 30 min. Transposed DNA fragments were purified using a ChIP DNA Clean & Concentrator Kit (Zymo Research). ATAC-seq libraries were prepared using Phusion High-Fidelity DNA Polymerase (NEB) in a 25 µL PCR reaction containing 12.5 µL 2 × Phusion Master Mix, 1.25 µL 10 mM Ad1 primer, 1.25 µL 10 mM Ad2 primer, 4 µL nuclease-free water, and 6 µL purified transposed DNA fragments. The final ATAC-seq libraries were sequenced on an Illumina HiSeq 2500 platform according to the manufacturer’s instructions. Adaptor sequences were removed from raw ATAC-seq reads using Trim Galore to ensure high-quality read processing. The cleaned reads were then aligned to the Wm82 soybean reference genome using Bowtie2 with the following parameters: -X 2000 -m 1, allowing for a maximum fragment length of 2000 bp and using only uniquely mapped reads (Langmead and Salzberg 2012). Reads originating from chloroplast and mitochondrial DNA were discarded to remove non-nuclear contamination, and duplicate reads were removed using Samtools (Li et al. 2009). The chromatin states were analyzed using ChromHMM with default settings (Ernst and Kellis 2017). BigWig format track files were generated using the bamCoverage tool of deeptools (v3.5.1) with RPKM normalization for metaplots (Ramirez et al. 2016). ChIP-seq peaks were identified using MACS2 (v2.1.1), and peak annotations (e.g., promoters, gene bodies, intergenic regions) were performed using ChIPseeker (Yu et al. 2015; Zhang et al. 2008).

### Hi-C analysis

Hi-C data from soybean leaves were downloaded from the NCBI Sequence Read Archive under accession numbers SRR12494514 and SRR12494515 (Wang et al. 2021). Paired-end Hi-C sequencing reads were aligned to the soybean Wm82 reference genome using HiC-Pro (v2.11.1) (Servant et al. 2015). Post-alignment, contact matrices were generated, and whole-genome Hi-C heatmaps were constructed using Juicer Tools (v1.13.02) and visualized in Juicebox (v1.11.08) (Durand et al. 2016). Hi-C visualization was performed by HiCExplorer (version 3.4.3) (Wolff et al. 2018). TADs were detected by the hicFindTADs function in HiCExplorer with 5000 bp-resolution and default parameters.

### RNA-seq analysis

RNA-seq data were obtained from published studies (Wang et al. 2023). RNA-seq reads were aligned to the soybean Wm82 reference genome using Bowtie2, ensuring high mapping accuracy and efficient transcript quantification (Langmead and Salzberg 2012). Gene expression levels were calculated as TPM (transcripts per million) using RSEM (v1.3.1) with default settings, employing rsem-the calculate-expression tool for estimation of transcript abundance and edgeR for DEG calling (Li and Dewey 2011). Processed RNA-seq data of soybean leaves under different stress conditions were obtained from a previous study (Yu et al. 2022). The correlation between RNA-seq and RT-qPCR validation was assessed by comparing the fold-change values between treatment and control conditions.

## Supplementary Information

Below is the link to the electronic supplementary material.

Supplementary file1 (XLSX 24 KB)

Supplementary file2 (DOCX 2298 KB)

## Data Availability

The high-throughput sequencing data generated in this study have been deposited in the National Center for Biotechnology Information database under accession number PRJNA1235221.
