# Supplementary material for: Epigenetically poised chromatin states regulate PRR and NLR genes in soybean
Source: aBIOTECH. 2025 Aug 8;6(3):411–23. doi: 10.1007/s42994-025-00233-4 (PMC12454805; doi:10.1007/s42994-025-00233-4)
Supplement: Supplementary file 2 — Supplementary file2 (DOCX 2298 KB) [file 42994_2025_233_MOESM2_ESM.docx]

**Epigenetically poised chromatin states regulate *PRR* and *NLR* genes in soybean**

Linzhe Jin^1^, Yihan Zhang^1^, Jiayuan Guo^1^, Xuexia Liu^1^, Yanling Lai^1^, Xinfang Huang^1^, Yuhan Zou^1^, Shichuan Yan^1^, Xianzhe Dai^1^, Zhenhui Zhong^1*^

^1^ Ministry of Education Key Laboratory for Bio-Resource and Eco-Environment, College of Life Sciences, State Key Laboratory of Hydraulics and Mountain River Engineering, Sichuan University, Chengdu 610064, China

* To whom correspondence may be addressed. E-mail: zhenhuizhong@scu.edu.cn


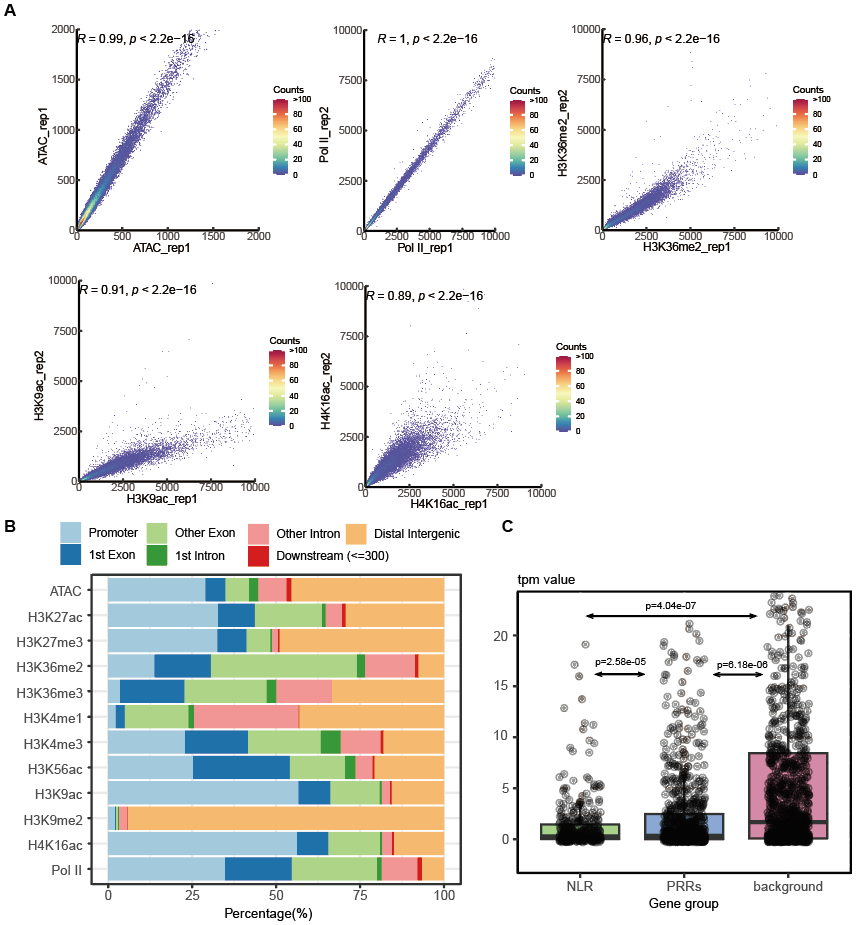


**Supplementary Fig. S1 A** Pearson correlation of two replicates of ATAC-seq and ChIP-seq (Pol II, H3K36me2, H3K9ac, and H4K16ac). **B** Peak annotation of different histone modifications, ATAC-seq, and Pol II peaks in soybean. **C** Expression level of PRRs and NLRs compared with random control


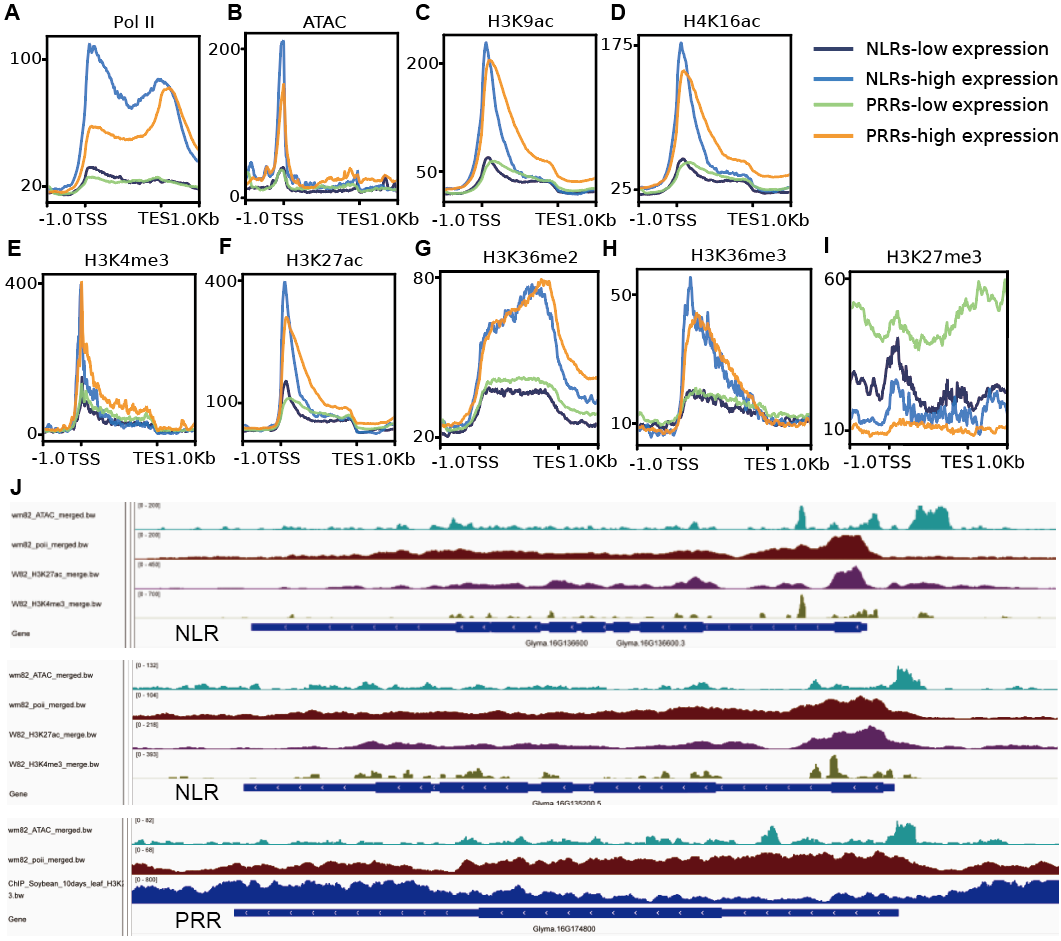


**Supplementary Fig. S2** Pol II ChIP-seq **(A)**, ATAC-seq **(B)**, H3K9ac ChIP-seq **(C)**, H4K16ac ChIP-seq **(D)**, H3K4me3 ChIP-seq **(E)**, H3K27ac ChIP-seq **(F)**, H3K36me2 ChIP-seq **(G)**, H3K36me3 ChIP-seq **(H)**, H3K27me3 ChIP-seq **(I)** data over NLR and PRR genes. Both NLR and PRR have been divided into two groups according to their expression level. **J** Examples of H3K27me3 enriched at 5’ end of NLR and flanking regions of PRR


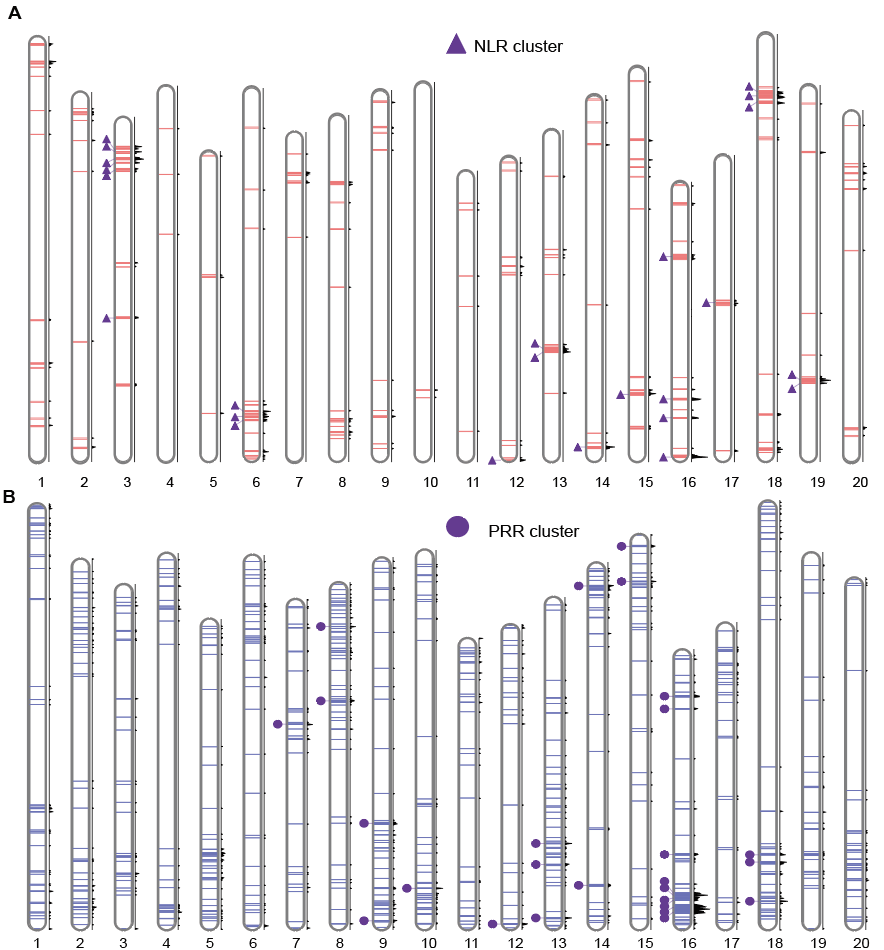


**Supplementary Fig. S3 A** Chromosomal distribution of NLRs in soybean Wm82 genome. The triangle stands for NLR clusters identified in this study. **B** Chromosomal distribution of PRRs in soybean Wm82 genome. The cycle stands for PRR clusters identified in this study


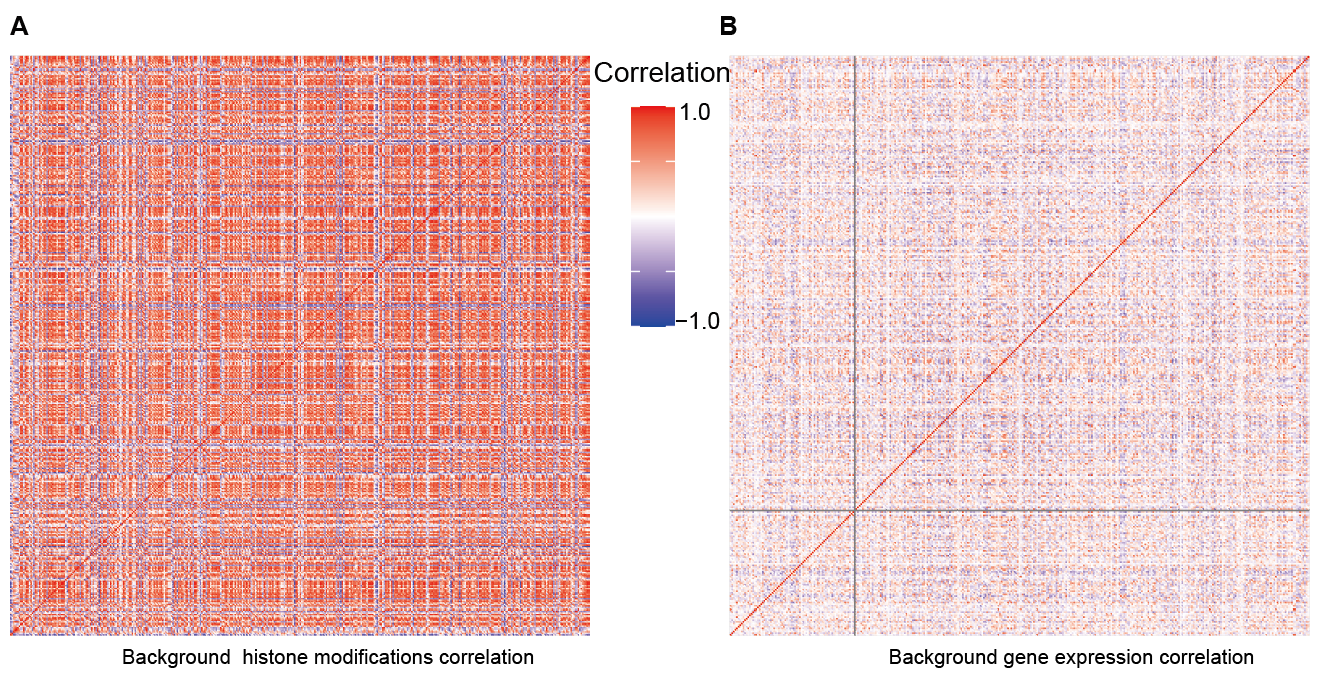
 **Supplementary Fig. S4** Pairwise Pearson correlation analysis of histone modifications (**A**) and gene expression levels (**B**) for random control


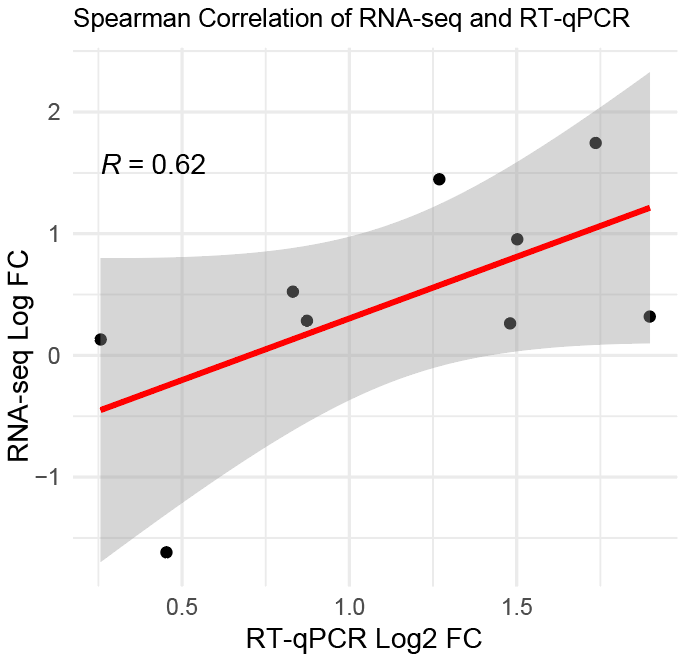


**Supplementary Fig. S5** Pairwise Spearman correlation analysis of RNA-seq data and RT-qPCR validation. Log2 fold change of RNA-seq (RPKM) and relative expression level quantified by RT-qPCR results were plot on y and x axis, respectively. qRT-PCR primers used in this study are presented in the Supplementary Table 2.
